# Highlighting the Importance of Preoperative Imaging in Neurocysticercosis: A Case Report of Rapid Cysticercus Cyst Migration From the Third to the Fourth Ventricle

**DOI:** 10.7759/cureus.83527

**Published:** 2025-05-05

**Authors:** John P Mader, Sepehr Zekavaty, James Yang, Ryan Rossi, Chad Thompson, Shamseldeen Y Mahmoud

**Affiliations:** 1 Radiology, Saint Louis University School of Medicine, Saint Louis, USA

**Keywords:** cysticercus, migrating cyst, mri, neurocysticercosis, taenia solium

## Abstract

Neurocysticercosis is a parasitic infection of the central nervous system commonly caused by *Taenia solium*. Imaging with magnetic resonance imaging (MRI) is critical for diagnosis and follow-up of the infection. In this report, we present the case of a 44-year-old male with a history of chronic neurocysticercosis who presented with acute neurological symptoms. An initial MRI showed new obstructive hydrocephalus due to a cyst in the third ventricle. The patient underwent placement of an external ventricular drain to relieve the obstructing hydrocephalus. Follow-up imaging revealed interval migration of the cyst to the fourth ventricle. The cyst was removed surgically, and the patient recovered without any complications. This case emphasizes the importance of timely imaging for effective surgical planning of neurocysticercosis.

## Introduction

Neurocysticercosis is a helminthic infection caused by *Taenia solium* that is often transmitted through the consumption of inadequately cooked pork. Involvement of the central nervous system (CNS) may occur and is associated with a variety of symptoms, including epilepsy, focal deficits, intracranial hypertension, and cognitive decline. The disease is endemic to developing countries in Central and South America, Sub-Saharan Africa, and Asia, often affecting low-income and rural communities with poor sanitation [1]. However, with increased immigration and mobility, there has been an increase in cases of neurocysticercosis in the developed world [2]. Neurocysticercosis has been designated a neglected tropical disease by the World Health Organization [3]. In the United States, between 2003 and 2012, it was estimated that there were 18,584 hospitalizations for neurocysticercosis with associated hospital charges over $900 million [3]. Neuroimaging, particularly magnetic resonance imaging (MRI), is crucial for the diagnosis and management of neurocysticercosis. This report presents a unique case of cysticercus cyst migration from the third to the fourth ventricle within days.

## Case presentation

**Figure 1:**
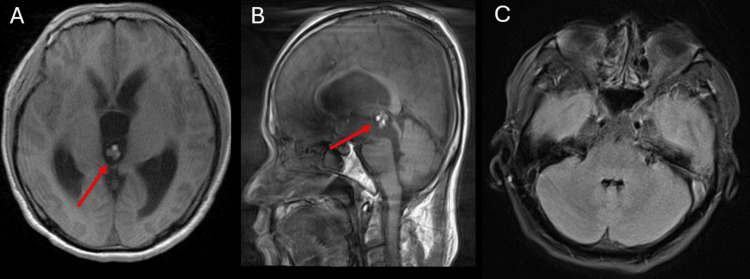
MRI of the brain with and without contrast on hospital day three. Significant motion artifact is present. A: Axial T1-weighted imaging shows ventriculomegaly and a heterogeneous cystic lesion in the third ventricle (red arrow). B: Sagittal T1-weighted imaging shows ventricular dilation and a heterogeneous cystic lesion in the third ventricle (red arrow). C: Axial T2 fluid-attenuated inversion recovery imaging shows non-dilated fourth ventricle and no evidence of cystic lesions.

In the summer of 2024, a 44-year-old male with chronic neurocysticercosis (initially diagnosed on imaging in 2022) presented to the hospital with vision changes, headache, abdominal pain, dizziness worsened with head movement, nausea, and vomiting. The patient’s new symptoms started during work at his factory job. He initially presented to an outpatient clinic and was advised to take a couple of days off work, but his headache did not resolve, and he went to the emergency department. Vitals and physical examination were unremarkable. Labs were significant for an elevated white blood cell count with increased absolute neutrophils, as well as elevated cysticercus antibody IgG. Creatinine, hemoglobin, lipase, B-type natriuretic peptide, troponin, ethanol, and a viral panel were normal (Table 1). A computed tomography (CT) scan was performed and revealed obstructive hydrocephalus, and the patient was transferred for neurosurgery evaluation. Brain MRI was performed on hospital day three for further investigation (Figures 1A-1C). Axial T1 imaging showed prominent ventriculomegaly with a cystic lesion in the third ventricle (Figure 1A). Sagittal T1 imaging showed dilated ventricles with a heterogenous cystic lesion (Figure 1B). Notably, the fourth ventricle remained non-dilated and did not show evidence of any cysts at this time (Figure 1C).

**Table 1:** The patient’s relevant laboratory findings with corresponding reference ranges. Abnormal values are in bold.

Lab	Reference range	Patient’s value
White blood cell count	4.0–10.7 × 10^9^/L	13.1 × 10^9^/L
Absolute neutrophil count	1.60–7.50 × 10^9^/L	10.07 × 10^9^/L
Creatinine	0.71–1.16 mg/dL	0.80 mg/dL
Hemoglobin	13.3–17.5 g/dL	13.6 g/dL
Lipase	13–75 U/L	25 U/L
B-type natriuretic peptide	0–99 pg/mL	<10 pg/mL
Troponin	0–0.03 ng/mL	<0.01 ng/mL
Alcohol	<10 mg/dL	<10 mg/dL
Viral panel	Negative	Negative
Cysticercus antibody IgG	<8 units	12 units
Echinococcus antibody IgG	<9 units	8 units

Neurosurgery placed an external ventricular drain on hospital day four, which led to improvement in obstructive hydrocephalus and clinical symptoms.

**Figure 2:**
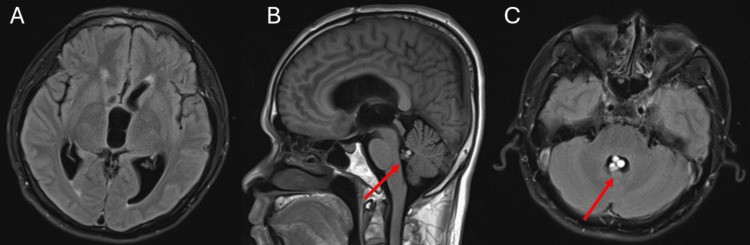
MRI of the brain on hospital day eight showing interval migration of cystic lesions from the third ventricle to the fourth ventricle. A: Axial T2 fluid-attenuated inversion recovery imaging shows persistent ventriculomegaly without evidence of the previously noted cyst in the third ventricle. B: Sagittal T1-weighted image shows ventricular dilation without evidence of the previously noted third ventricular cyst. C: Axial T1-weighted imaging shows a migrated cystic lesion now in the fourth ventricle.

**Figure 3:**
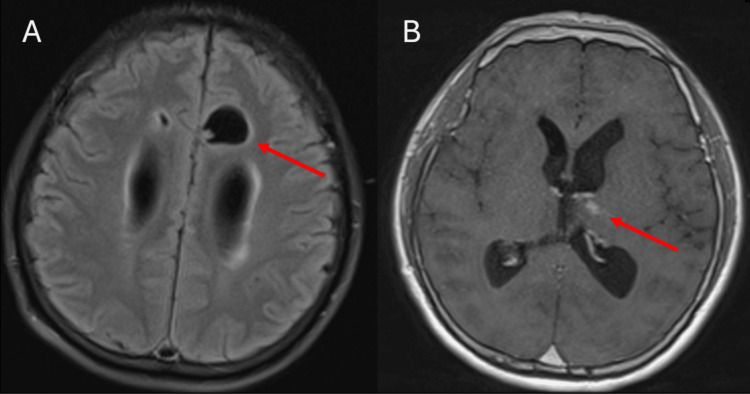
MRI of the brain on hospital day eight. A: Axial T2 fluid-attenuated inversion recovery imaging shows a 2.1 cm dominant cystic lesion with a small peripheral solid nodule, along the parasagittal left frontal lobe concerning for the active vesicular stage of the disease. B: Axial T1 post-contrast imaging shows one of several small areas of parenchymal enhancement in the left thalamus concerning for active disease.

Per Infectious Diseases Society of America guidelines, there was an operative plan to remove the cysticercus cysts without preoperative antiparasitic therapy [4]. A repeat MRI was obtained on hospital day eight to guide surgical planning, which revealed interval migration of the cysts from the third to the fourth ventricle (Figures 2A-2C). Additionally, a parasagittal predominantly cystic lesion with a small nodule in the left frontal lobe was noted (Figure 3A). These findings were previously identified on the initial MRI on admission and were concerning for the active vesicular stage of the disease. Axial imaging also identified a small focus of enhancement in the left thalamus, which further supported an active disease process (Figure 3B).

**Figure 4:**
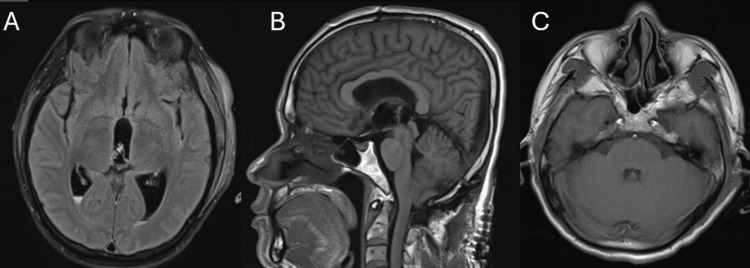
MRI of the brain on hospital day 12 following the removal of a 1.5 cm cyst from the fourth ventricle. A: Axial T2 fluid-attenuated inversion recovery imaging showing small-volume postoperative intraventricular hemorrhage without evidence of residual cysts. B: Sagittal T1-weighted image showing no evidence of residual cysts. C: Axial T1 image showing no evidence of residual intraventricular cysts (Figure 2).

**Figure 5:**
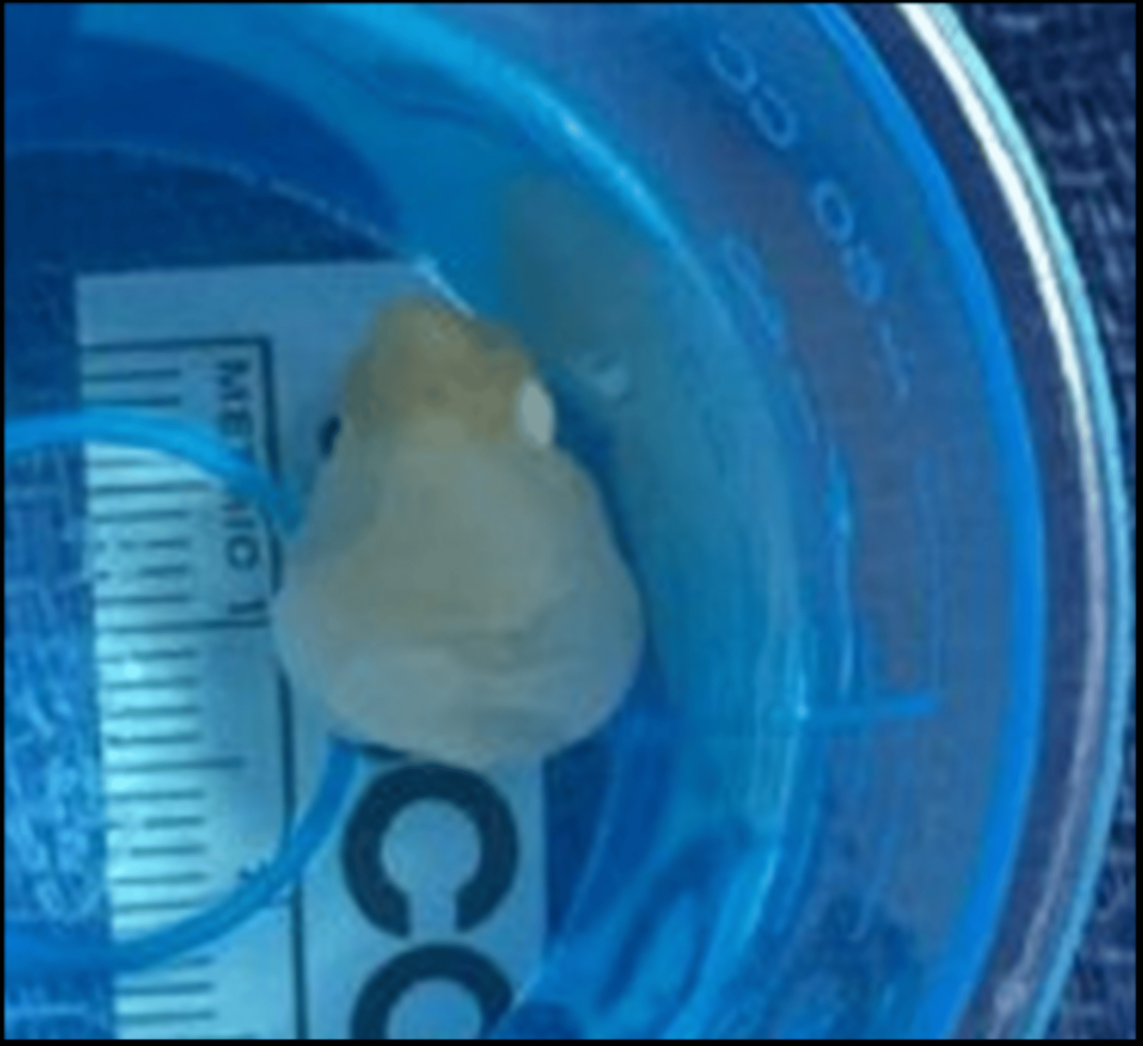
Gross specimen of the 1.5 cm cyst that was removed from the fourth ventricle. The specimen is seen inside the examination cup.

On hospital day 12, the patient underwent surgical removal of the fourth ventricular cyst. Postoperative brain MRI showed successful cyst removal with no residual ventricular cysts (Figures 4A-4C). Figure 5 shows the gross pathology of the removed cyst. After the procedure, the patient was started on a 14-day course of albendazole 600 mg every 12 hours and praziquantel 1,200 mg every eight hours. His symptoms improved, and he was discharged home with appropriate follow-up.

## Discussion

Intraventricular cysts in neurocysticercosis occur when *Taenia solium* larvae reach the brain’s ventricular system, most likely through the choroid plexus [5]. These cysts may go unnoticed until they block cerebrospinal fluid (CSF) pathways and cause symptoms such as nausea, vomiting, headache, decreased visual acuity, altered mental status, and cranial nerve palsies [5]. To effectively diagnose and manage neurocysticercosis, neuroimaging and serology are required to determine the type and stage of the disease. At a fundamental level, lesions can be categorized as parenchymal or extraparenchymal [1].

Differential diagnoses for cysticercus cysts on imaging can include tuberculosis, toxoplasmosis, malignancy, or pyogenic cerebral abscess [6]. A *ventricular migration sign*, in which the cystic structures migrate between ventricles, has been known to increase the likelihood of a diagnosis of ventricular cysticercosis, as other potential causes would not show the same type of migration [2]. The mobility of the cysts is generally caused by head movement [2]. This migration can lead to the obstruction of CSF flow and result in hydrocephalus [7].

Migration of ventricular cysts has been reported in several case studies [8]. This movement is important because it can change the surgical approach. Cysts commonly migrate to the fourth ventricle, but they can shift between any of the ventricles [8]. Thus, the dynamic nature of this phenomenon highlights the need for updated imaging during preoperative planning.

The patient’s MRI from hospital day three (Figure 1) showed cysts within the third ventricle. The MRI from hospital day eight showed cysts within the fourth ventricle (Figure 2). In a matter of mere days, the patient’s cysts had moved significantly, which would have complicated their surgical removal had the follow-up MRI on hospital day eight not been obtained. This highlights the importance of imaging as close to the surgical procedure as possible to give the procedure the highest likelihood of success. Although ventricular cysts are not seen as clearly on CT imaging, their position can still be elucidated on CT, and it could be used as an alternative to MRI to assess cyst migration if obtaining another pre-surgical MRI is not possible.

This study’s limitations include a lack of generalizability, as it is a single case report. In addition, cyst migration was observed and influenced surgical planning; however, the prevalence and significance of this phenomenon require larger studies to investigate.

## Conclusions

Understanding the chronology of disease states for the differential diagnoses of a patient presenting with cystic structures of the brain is important for radiologists. Migration of cysts can present an issue for planning surgical removal. This case highlights the importance of interval imaging for the removal of cysts in a patient with neurocysticercosis.
